# The Thirty-Ninth Annual Commencement of the Baltimore College of Dental Surgery

**Published:** 1879-03

**Authors:** 


### EDITORIAL, ETC.

*The Thirty-Ninth Annual Commencement of the Baltimore College of Dental Surgery* was held at the Academy of Music, on the evening of March 6th, 1879, before a large and brilliant audience, composed in a great part of ladies, as is usual on such occasions. The Faculty of the College, many of the Alumni and the Graduates, occupied the stage together with a number of others distinguished in the dental and medical professions. The legal profession was represented by the Hon. Judge George Wm. Brown, of Baltimore.

The Fifth Regiment Orchestra, Professor Itzell, director, rendered a number of fine selections during the evening

The exercises were opened with prayer by the Rev. Julius E. Grammer, D. D., rector of St. Peter's Church, Druid Hill Avenue.

The announcement of graduates was made by Prof. F. J. S. Gorgas, Dean, embracing the following, forty-one in number:

| | Subject of Thesis. |
|---|---|
| A. M. I. Hein, | *Germany.*—The Teeth. |
| A. H. Grieffenhagen, | *Germany.*—Extraction of Teeth. |
| Samuel Edward Jones, | *Texas.*—Dental Caries. |
| Frank Pearce Bernard, | *Pennsylvania.*—Special Senses. |
| William Rust Laws, | *Maryland.*—Irregularity of the Teeth. |
| Juan Bautista Lombard, | *Cuba.*—Stomatitis. |
| Edwardo Lombard, | *Cuba.*—Aneurisms of the Tongue. |
| Bolivar J. Quattlebaum, | *South Carolina.*—Alveolar Abscess. |
| William Roberts Renalds, | *Virginia.*—Dental Caries. |
| James Armstead Colvin, | *Virginia.*—First Dentition. |
| Laurence Stafford Wolfe, | *S. Carolina.*—Stomatitis. |
| Charles M. N. Latimer, | *Dist. Columbia.*—The Teeth of Man. |
| Andreas C. Ferrari, | *Russia.*—Treatment of Dental Pulp. |
| J. Allen Patterson, | *South Carolina.*—Treatment of Dental Pulp. |
| Gordon White, | *Tennessee.*—Neuralgia. |
| Frank Brumit Perry, | *Virginia.*—Treatment of Dental Pulp. |
| Arthur Monroe Rice, | *Connecticut.*—Treatment of Dental Pulp. |

| | | |
|---|---|---|
| John C. Wilkerson, | *Alabama.*— | Dental Caries. |
| Robert P. Fletcher, | *Virginia.*— | Treatment of Dental Pulp. |
| John Madison Phillips, | *N. Carolina.*— | Treatment of Dental Pulp. |
| Richard Hance Billingsley, | *Maryland.*— | Maxillary Sinus. |
| Thomas Sadler Jordan, | *Alabama.*— | Dental Hygiene. |
| William M. Rawlinson, | *South Carolina.*— | Filling Teeth. |
| Thomas G. Morrow, | *Maryland.*— | Circulation of Blood. |
| Richard Grady, | *Maryland.*— | Treatment of Pulp. |
| John P. Coult, | *New Jersey.*— | Treatment of Pulp. |
| Charles Eckhardt, | *Lousiana.*— | Odontalgia. |
| William H. Weems, | *Texas.*— | Treatment of Pulp. |
| Francis S. Harris, | *North Carolina.*— | The Care of Children's Teeth. |
| Edwin D. Akers, | *Pennsylvania.*— | Inflammation of Gums. |
| T. Disbrow Stone, | *New York.*— | Caries. |
| Elroy F. Cross, | *Massachusetts.*— | Treatment of Pulp. |
| Edmund C. Bryant, | *Maine.*— | Treatment of Pulp. |
| Edwin J. Parkison, | *Maryland.*— | Salivary Calculus. |
| Alfred F. King, | *New York.*— | The Six-Year Molars |
| Hall Lewis, | *District Columbia.*— | Treatment of Pulp. |
| Samuel M. Roach, | *Georgia.*— | Filling Teeth. |
| Edward Flannigain, | *Maryland.*— | Artistic Dentistry. |
| Richard B. Winder, Jr, | *Maryland.*— | |
| John A. Millard, | *France.*— | |
| Henry Bentley, | *Connecticut.*— | |

Sixteen of the United States were represented, and also Germany, France, Russia and Cuba.

The degree of "Doctor of Dental Surgery," was then conferred upon the above named Students by Prof. F. J. S. Gorgas, Dean of the College. Before conferring the degrees the Dean announced the union of the Maryland Dental College with the Baltimore College of Dental Surgery, under the Charter of the latter; and also the election of Professor Richard B. Winder, D. D. S., M. D., to the chair of "Dental Surgery." The Faculty as now constituted is as follows:

FERD. J. S. GORGAS, A. M., M. D., D. D. S., Professor of Pathology and Therapeutics.

E. LLOYD HOWARD, M. D., Professor of Chemistry and Materia Medica.

JAMES H. HARRIS, M. D., D. D. S., Professor of Clinical Dentistry.

JAMES B. HODGKIN, D. D. S., Professor of Dental Mechanism and Metallurgy.

THOS. S. LATIMER, M. D., Professor of Anatomy and Physiology.

RICHARD B. WINDER, D. D. S., M. D., Professor of Dental Surgery.

GEO. H. CHEWNING, D. D. S., Demonstrator of Operative Dentistry.

AUGUSTUS W. SWEENY, JR., D. D. S., Assistant Demonstrator.

John C. Uhler, D. D. S., M. D., Demonstrator of Mechanical Dentistry.
Luke J. Pearce, D. D. S., Assistant Demonstrator.
Charles F. Bevan, M. D., Demonstrator of Anatomy.

The Valedictory Address was delivered by Rev. Alex. W. Weddell, D. D., of Richmond, Va., who, in his interesting and highly appreciated remarks, alluded to the origin of the valedictory, and said that after his own experience as a graduate, listening to a valedictorian for two solid hours in the heat of a July sun, he could appreciate the graduates' feelings when waiting to receive the smiles and congratulations of their many fair friends, and would hail with cordial support a seventeenth amendment prohibiting their appearance forever on American soil. The Baltimore College was highly eulogized by the speaker, who touched upon the various courses of study, and said that each one of the graduates on this occasion had a solid foundation on which to build their super-structure. "Those who had left this, the oldest institution in the country, and who now numbered nearly 900, have proved to the world that the work of the College has not been misjudged."

"The Dean of the Faculty in placing in your hands, young graduates, the diploma of the college, has placed among these other names your own, and it rests upon you to see that the honor is well sustained. The woes of humanity, like the wounds of Cæsar, are voiced in prayer for help. To such prayer your hearts lend a responsive ear. The deeper your insight in the evil threatened and into the evil existing, the greater will be your determination to toil after the best preventive and the most certain cure, and your professional pride, saturated with generous sympathies, will render you liberal, and such a life can never know the shame of failure."

This address will appear in the April number of the Journal.

The Class Address was delivered by Frank B. Perry, of Virginia, of the graduating class, and was a well prepared oration, fluently spoken.

The graduates were the recipients of many beautiful bouquets on receiving their diplomas.

The benediction, by the Rev. Dr. Grammer, closed the exercises at the Academy.

The number of matriculates for the session just closed, was eighty-two.

The Fortieth Annual Session will commence on the 15th of October, 1879, and continue until March, 1880.

